# Mutant Ubiquitin Attenuates Interleukin-1β- and Tumor Necrosis Factor-α-Induced Pro-Inflammatory Signaling in Human Astrocytic Cells

**DOI:** 10.1371/journal.pone.0067891

**Published:** 2013-07-03

**Authors:** Kyungsun Choi, Junseong Park, Jungsul Lee, Eun Chun Han, Chulhee Choi

**Affiliations:** 1 Cell Signaling and BioImaging Laboratory, Department of Bio and Brain Engineering, KAIST, Daejeon, Korea; 2 KI for the BioCentury, KAIST, Daejeon, Korea; 3 Graduate School of Medical Science and Engineering, KAIST, Daejeon, Korea; National Institute of Allergy and Infectious Diseases - Rocky Mountain Laboratories, United States of America

## Abstract

A frameshift mutation of ubiquitin called ubiquitin^+1^ (UBB^+1^) was found in the aging and Alzheimer’s disease brains and thought to be associated with neuronal dysfuction and degeneration. Even though ubiquitylation has been known to regulate vital cellular functions mainly through proteasome-dependent degradation of polyubiquitinated substrates, proteolysis-independent roles of ubiquitylation have emerged as key mechanisms in various signaling cascades. In this study, we have investigated the effect of UBB^+1^ on proinflammatory signaling such as interleukin-1β (IL-1β) and tumor necrosis factor-α (TNF-α) in human astrocytes. Treatment with TNF-α and IL-1β induced expression of CCL2 and CXCL8 by human astrocytic cells; while ectopic expression of UBB^+1^ significantly abrogated the proinflammatory cytokine-induced expression of chemokines. Ectopic expression of UBB^+1^ suppressed TNF-α- and IL-1β-induced activation of NF-κB and JNK signaling pathway. Furthermore, we have demonstrated that polyubiquitylation of TRAFs and subsequent phosphorylation of TAK1 were significantly inhibited by stable expression of UBB^+1^. Collectively, these results suggest that UBB^+1^ may affect proinflammatory signaling in the central nervous system via inhibitory mechanisms of ubiquitin-dependent signaling in human astrocytes.

## Introduction

Ubiquitylation has been well characterized to regulate vital cellular processes mainly through proteasome-dependent degradation of polyubiquitinated substrates; however, proteolysis-independent roles of ubiquitylation have emerged as key mechanisms in various signaling cascades [1], [2]. Typically, polyubiquitin chains that target proteins for degradation by the proteasome are linked through K48 of ubiquitin. On the contrary, K63-linked polyubiquitin chains play multiple roles in kinase activation, DNA repair and intracellular trafficking via proteasome-independent mechanisms [3], [4].

A frameshift mutation of ubiquitin called ubiquitin^+1^ (UBB^+1^) was found in the aging and Alzheimer’s disease (AD) brains [5]–[7]. UBB^+1^ is generated by transcriptional dinucleotide deletion within the mRNA resulting in a 19-amino acid extension at the C-terminus of ubiquitin [5]. This mutant ubiquitin cannot link to substrates targeted for proteasomal degradation, but is ubiquitylated to form a polyubiquitin chain. Ubiquitylated UBB^+1^ is refractory to deubiquitination, resulting in dominant inhibition of the ubiquitin-proteasome system (UPS) [7]–[9]. Recent evidences have revealed that UBB^+1^ is detected as pathological hallmarks in various neurodegenerative diseases and exacerbates the proteasomal dysfunction and deposition of toxic proteins [9]–[11]. It was also reported that UBB^+1^ exerts a neurotoxic effect by suppressing proteasome-dependent proteolysis in neurons [12]. Although UBB^+1^ can be found in non-neuronal cells [7], [13], [14], its functional significance has not yet been fully determined.

Astrocytes, the most abundant glial cells in the central nervous system (CNS), play important roles in maintaining the homeostatic environment and immune regulation, producing a repertoire of inflammatory mediators including chemokines, cytokines and adhesion molecules [15], [16]. Interleukin-1β (IL-1β) and tumor necrosis factor-α (TNF-α) serve as major regulators of immune and inflammatory responses in the CNS, and elevated expression of these cytokines occurs in injury, infection, stroke, inflammation and degenerative disorders such as AD [17], [18]. These proinflammatory cytokines induce expression of multiple genes associated with inflammation by human astrocytes [19]. In response to IL-1β and TNF-α, ubiquitylation-dependent activation of TNF-associated factor (TRAF) 6 and TRAF2 complexes leads to activation of TGF-β-activated kinase 1 (TAK1) which activates nuclear factor kappa B (NF-κB) and c-Jun NH_2_-terminus kinase (JNK) pathways [20], [21]. In this study, we investigated the effect of UBB^+1^ on proinflammatory signaling such as IL-1β and TNF-α in human astrocytes, and its functional relevance of ubiquitin-dependent kinase activation.

## Materials and Methods

### 1. Cell Culture

Human astrocytoma CRT-MG cells [22], [23] were maintained in RPMI 1640 medium that was supplemented with 2 mmol//L L-glutamine, 100 U/ml penicillin, and 100 g/L streptomycin and 10% heat-inactivated fetal bovine serum in a 5% CO_2_ incubator at 37°C.

### 2. Stable UBB^+1^ Cell Lines

For generation of the pEGFP-UBB^+1^ construct, the UBB^+1^ open reading frame was amplified by PCR from the pTet-Splice-UB plasmid and cloned in the *Eco*RI and HindIII sites of the EGFP-N1 vector (Clonetech, Palo Alto, CA). Stable cell lines transfected with the pEGFP or pEGFP-UBB^+1^ were generated. CRT-MG cells were transfected by electroporation (Amaxa Biosystems, Cologen, Germany) according to manufacturer’s instructions. Stable transfectants were grown in medium containing 0.5 g/L G-418 (Life Technologies, Carlsbad, CA) and cloned. Stable clonal cells were sorted by flow cytometry (Becton Dickinson, Mountain View, CA) based on GFP fluorescence intensity.

### 3. Reagents

Human recombinant IL-1β and TNF-α were purchased from R & D system (Minneapolis, MN, USA). Antibodies against TRAF2/6 and β-actin were purchased from Santa Cruz Biotechnology (Santa Cruz, CA). Antibodies specific for IKK, phospho-IKKα/β (Ser176/180), IκBα, phospho-IκBα (Ser32/36), MKK4, phospho-MKK4 (Ser257/Thr261), JNKs (p46 and p54), phospho-JNKs (Thr183/Try185), c-Jun, phospho-c-Jun (Ser73), ERK, phospho-ERK, TAK1 and phospho-TAK1 were purchased from Cell Signaling Biotechnology (Bevery, MA, USA). Antibodies specific for EGFP, ubiquitin and UBB^+1^ were obtained from AbFrontier (Seoul, Korea).

### 4. ELISA

Concentrations of CXCL8 (a.k.a., IL-8) and CCL2 (a.k.a., MCP-1) in the supernatants were determined using dual-antibody solid phase ELISA (Biosource International, Camarillo, CA). Each chemokine expression was normalized by the total amount of protein.

### 5. Measurement of DNA Binding Activity of NF-κB and AP-1

Nuclear extracts were obtained as described previously [22]. Nuclear extracts (5 µg) were assayed for the binding activity of p65/c-Rel (NF-κB) and c-jun (activator protein-1, AP-1) using the TransAM NF-κB and AP-1 assay kit (ActiveMotif, Carlsbad, CA, USA) according to the manufacturer’s manual.

### 6. Western Blotting and Immunoprecipitation

Cell lysates were electrophoresed in 10% SDS-PAGE gels and then transferred to nitrocellulose and probed with anti-phospho-IKK, IKK, phospho-IκBα, IκBα, phospho-MKK4, MKK4, phospho-JNK, JNK, phospho-c-Jun, c-Jun, TAK1, phospho-TAK1 and β-actin. The blots were developed by chemiluminescence (AbFrontier). Cell lysates (500 µg) were immunoprecipitated with 1 µg of anti-TRAF2 antibody (Santa Cruz) for 12 h at 4°C, followed by an additional 3 h incubation with protein A/G beads (Pierce, Rockford, IL, USA). The immunocomplexes were analyzed by 8% SDS-PAGE gels and immunoblotted with ubiquitin and TAK1.

### 7. Statistical Analysis

Data are presented as mean ± SD. Levels of significance for comparisons between samples were determined using Student’s *t*-test distribution.

## Results

### 1. Effect of UBB^+1^ on IL-1β- and TNF-α-induced Chemokine Expression

**Figure 1:**
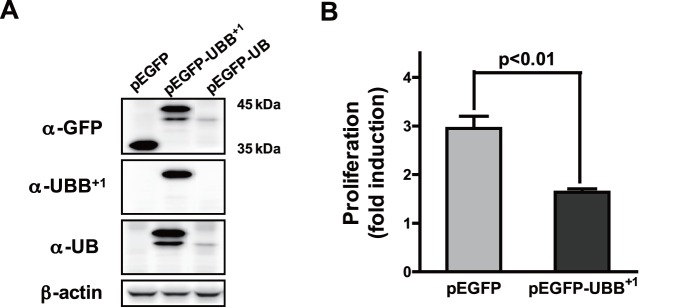
Ectopic expression of UBB^+1^ in human astrocytic cell. (A) CRT-MG cells were stably transfected with pEGFP, pEGFP-UBB^+1^ or pEGFP-UB constructs. Cell lysates were examined for EGFP, UBB^+1^ or wild-type UB protein by immunoblot analysis. (B) Cells stably transfected with pEGFP or pEGFP-UBB^+1^ constructs were plated on 60 mm^2^ dishes (3×10^5^ cells). After two days of culture, total cell number was counted after trypsinization. Proliferation rate was calculated as the fold induction of final cell number compared to the original cell number. Data are the mean ± SD for quadruplicate samples from two independent experiments.

To address the effect of UBB^+1^ on astrocytic functionality, we generated CRT-MG cells stably transfected with pEGFP or pEGFP-UBB^+1^ as described in materials and methods (Fig. 1A). Immunoblot using anti-UBB^+1^ antibody, specific to the 19 amino acid C-terminal epitope of UBB^+1^ revealed a definite increase in UBB^+1^ proteins in the cells transfected with pEGFP-UBB^+1^. These cells stably expressing UBB^+1^ showed significantly lower growth rates compared to the control cells (Fig. 1B).

**Figure 2:**
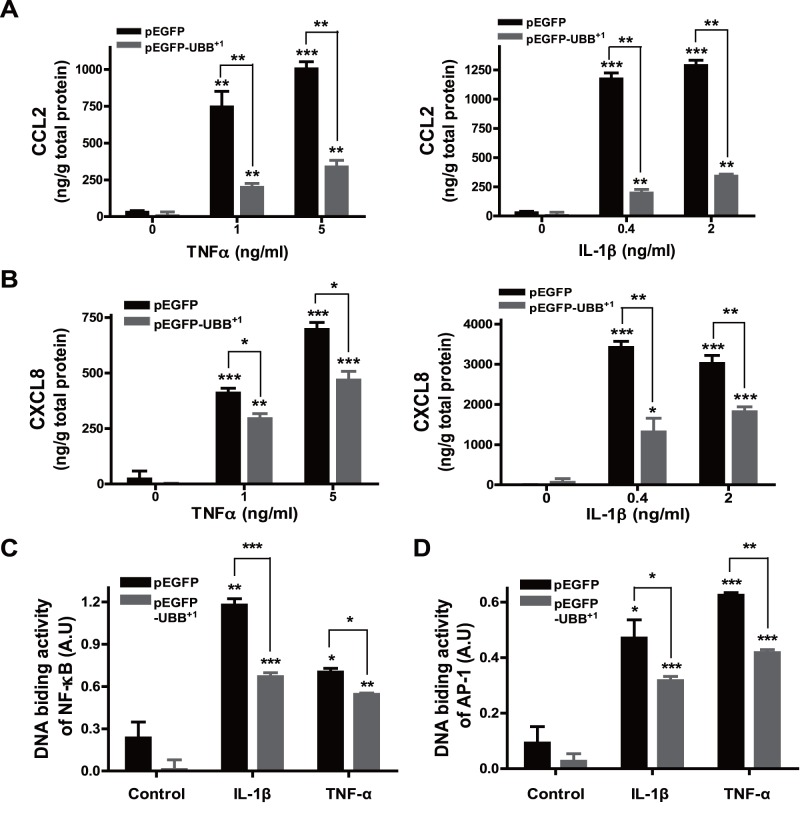
Effect of UBB^+1^ on IL-1β- and TNF-α-mediated chemokine expression. CRT-MG cells stably expressing pEGFP or pEGFP-UBB^+1^ were treated with varying doses of TNF-α or IL-1β for 24 h, and the supernatant was assayed for CCL-2 (A) or CXCL-8 (B) protein by ELISA. Control samples are the supernatants from the cells without any treatment. Representative of two independent experiments. Cells stably expressing pEGFP or pEGFP-UBB^+1^ were treated with IL-1β (4 µg/L) or TNF-α (10 µg/L) for 30 min, and then the nuclear extracts from cells were assayed for the binding activity of p65/c-Rel (NF-κB) (C) or c-Jun (AP-1) (D). Values statistically significantly different from the value for the control samples without any treatment are indicated by asterisks (*, p<0.05; **, p<0.01; and ***, p<0.001). Representative of two independent experiments.

To delineate the effect of UBB^+1^ on inflammatory response in astrocytes, we examined the expression of chemokines as a functional read-out for astroglial activation. In cells stably expressing pEGFP, treatment with IL-1β or TNF-α induced a robust expression of CCL2 and CXCL8 in a dose-dependent manner, which was dramatically attenuated by stable expression of UBB^+1^ (Fig. 2A & 2B). Since the expression of chemokines is known to be mainly regulated by activation of transcription factors such as NF-κB and AP-1 [24], [25], we next check the effect of UBB^+1^ on IL-1β- and TNF-α-induced activity of these transcription factors using a modified electrophoretic mobility shift assay as described in materials and methods. In control cells, treatment with IL-1β and TNF-α markedly induced the DNA binding activity of NF-κB and AP-1, which was also significantly reduced by UBB^+1^ expression (Fig. 2C & 2D).

### 2. Inhibitory Effect of UBB^+1^ Expression on NF-κB and JNK Pathway

**Figure 3:**
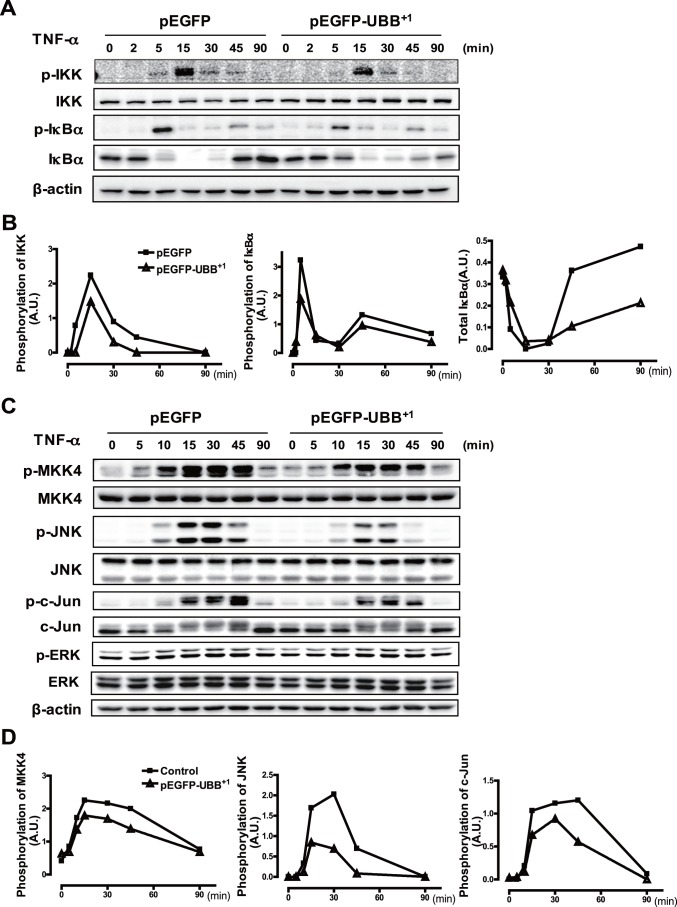
Effect of UBB^+1^ on the NF-κB and JNK pathway. (A) CRT-MG cells stably expressing pEGFP or pEGFP-UBB^+1^ were treated with TNF-α (10 µg/L) for the indicated time periods. Cell lysates were examined by immunoblot analysis with antibodies to phospho-IKK, IKK, phospho-IκBα, IκBα and β-actin. (B) The relative amount of phosphorylated IKK, phosphorylated IκBα and total IκBα was plotted over the time upon TNF-α treatment in pEGFP-UBB^+1^ stable cells (dotted line) and pEGFP stable cells (solid line). Ratiometric measurement of each blot was normalized by the value of β-actin. Representative of four independent experiments. (C) Cells stably expressing pEGFP or pEGFP-UBB^+1^ were treated with TNF-α (10 µg/L) for the indicated time periods. Cell lysates were examined by immunoblot analysis with antibodies to phospho-MKK4, MKK4, phospho-JNK, JNK, phospho-c-Jun, c-Jun, phospho-ERK1/2, ERK1/2 and β-actin. (D) The relative amount of phosphorylated MKK4, phosphorylated JNK and phosphorylated c-Jun was plotted over the time upon TNF-α treatment in pEGFP-UBB^+1^ stable cells (dotted line) and pEGFP stable cells (solid line). Ratiometric measurement of each blot was normalized by the value of β-actin. Representative of four independent experiments.

We next investigated the effect of stable UBB^+1^ expression on TNF-α-induced NF-κB signaling pathway by immunoblot analysis. It has been reported that activity of NF-κB is regulated by IκB kinase (IKK)-mediated phosphorylation and subsequent proteasomal degradation of IκBα leading to nuclear translocation of the NF-κB complex [26]. Treatment with TNF-α induced IκBα phosphorylation within 5 min of stimulation and subsequent degradation of IκBα by 15 min, which was followed by delayed expression of IκBα due to NF-κB-dependent IκBα production. Consistent with this, treatment with TNF-α enhanced IKK phosphorylation in the control cells in a time-dependent manner (Fig. 3A). Phosphorylation of IKK and IκBα was dramatically reduced in UBB^+1^ stable cells. In addition, the proteolytic degradation and de novo synthesis of IκBα were also decreased by ectopic UBB^+1^ expression (Fig. 3A).

Next, we examined the effect of UBB^+1^ on upstream signaling pathway involved in TNF-α-induced AP-1 activation. It has been shown that activity of AP-1 is regulated by the stress-activated protein kinases (SAPKs) cascades including JNK, which is activated by upstream kinases such as mitogen-activated protein kinase kinase (MKK) 4 [27], [28]. In the control cells, treatment with TNF-α induced phosphorylation of MKK4, which in turn was followed by phosphorylation of JNK and c-Jun in a time-dependent manner. Consistent with NF-κB results, phosphorylation of JNK pathway proteins by TNF-α was also attenuated by UBB^+1^ expression. On the contrary, TNF-α-induced phosphorylation of ERK was not affected by ectopic expression of UBB^+1^. The inhibitory effect of UBB^+1^ was also confirmed in IL-1β-treated cells (data not shown). These results collectively indicate that UBB^+1^ inhibited the NF-κB and JNK-AP-1 pathways but not the ERK pathway (Fig. 3B).

### 3. Inhibitory Effect of UBB^+1^ on Polyubiquitylation of TRAF Proteins and Subsequent TAK1 Phosphorylation

**Figure 4:**
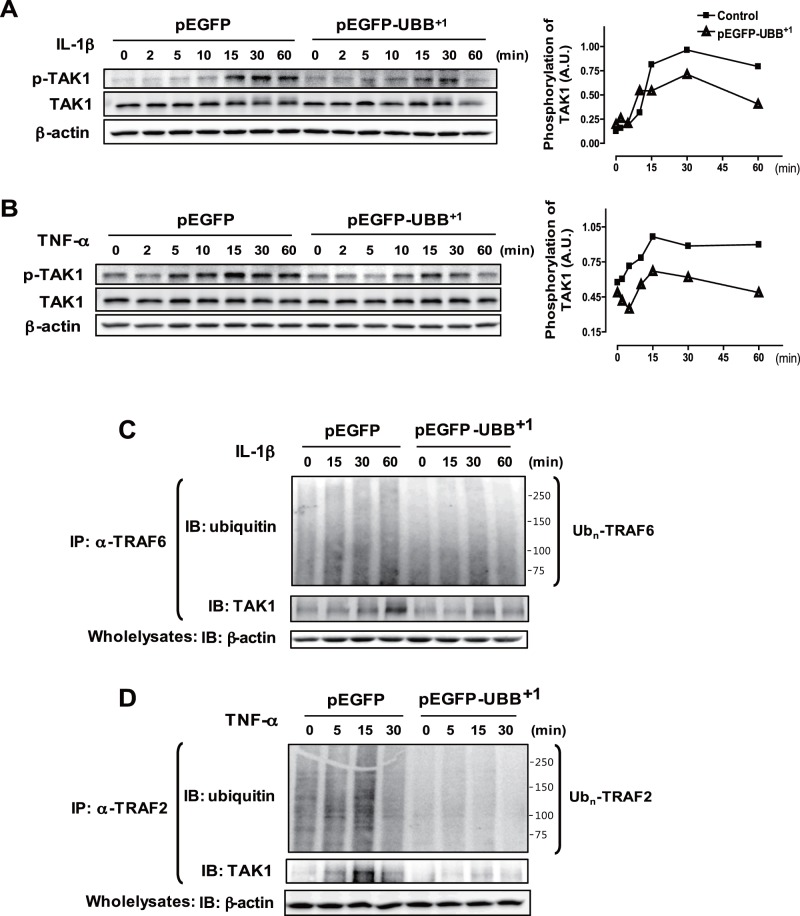
Inhibitory effect of UBB^+1^ on IL-1β- and TNF-α-induced TAK1 phosphorylation by polyubiquitylation of TRAF6 and TRAF2. CRT-MG cells stably expressing pEGFP or pEGFP-UBB^+1^ were treated with IL-1β (A) or TNF-α (B) for the indicated time periods. Cell lysates were examined by immunoblot analysis with antibodies to phospho-TAK1, TAK1 and β-actin. The relative amount of phosphorylated TAK1 was plotted over the time upon IL-1β and TNF-α treatment in pEGFP-UBB^+1^ stable cells (triangle) and pEGFP stable cells (square). Representative of three independent experiments. (C) Cells were treated with IL-1β for the indicated time periods, and then lysates were precipitated with anti-TRAF6 and blotted with anti-Ub. The whole cell lysates were subject to immunoblot analysis with antibodies to TAK1 and β-actin. Smear of high molecular weight proteins by ubiquitin blots represent poly-ubiquitylated TRAF6. (D) Cells were treated with TNF-α for the indicated time periods, and then lysates were precipitated with anti-TRAF2 and blotted with anti-Ub. The whole cell lysates were examined by immunoblot analysis with antibodies to TAK1 and β-actin. Smear of high molecular weight proteins by ubiquitin blots represent poly-ubiquitylated TRAF2. Representative of two independent experiments.

TAK1 has been identified as a key regulator of IKK and JNK pathway leading to the activation of NF-κB and AP-1 transcription factors in response to proinflammatory cytokines [29]. As expected, TAK1 was phosphorylated upon IL-1β or TNF-α stimulation in a time-dependent manner, which was significantly, attenuated in UBB^+1^ stable cell lines (Fig. 4A & B). TRAF proteins are signal transducers that mediate IL-1β- and TNF-α-mediated activation of TAK1 through K63-polyubiquitylation [8], [29]. As shown in Fig. 4C, a smear of high molecular weight proteins representing ubiquitylated TRAF6 increased upon IL-1β treatment, whereas these ubiquitylated form of TRAF6 was significantly reduced in UBB^+1^ stable cells. Consistent with this, TRAF6-TAK1 interactions were significantly enhanced upon IL-1β treatment, whereas these interactions between ubiquitylated TRAF6 and TAK1 were significantly reduced in UBB^+1^ stable cells. TNF-α-induced ubiquitylation of TRAF2 and interaction with TAK1 were also inhibited by UBB^+1^ expression (Fig. 4D). These results collectively indicated that UBB^+1^ potently abrogated ubiquitin-dependent TAK1 activation and subsequent activation of NF-κB and JNK pathway.

## Discussion

In this report, we have demonstrated that UBB^+1^ may affect proinflammatory signaling in the CNS via inhibitory mechanisms of ubiquitin-dependent signaling in human astrocytes. To our best knowledge, it is the first study of the potential role of UBB^+1^ on the inflammatory function in the astrocytes, with an emphasis on IL-1β and TNF-αsignaling.

The accumulation of UBB^+1^ has been regarded to represent the proteasomal dysfunction in the diseased brain [30]. The levels of UBB^+1^ protein proceeded functional impairment of the UPS in a dose-dependent manner, shifting from a proteasome substrate to a reversible inhibitor of the UPS. UBB^+1^ can also be degraded by the proteasome via internal K29- or K48-linked polyubiquitination at low levels of expression; however, at higher concentration, UBB^+1^ can act as a reversible antagonist of the UPS [8], [9].

It has been increasingly appreciated that ubiquitylation of the UBB^+1^ can also occur at the internal residue of K63, which gives rise to K63 polyubiquitin chains [31], [32]. The K63 ubiquitylation has been proposed to regulate various cellular signaling through non-proteolytic mechanisms [3], [33]. Thus we hypothesized that ubiquitinated UBB^+1^ can alter cell homeostasis and signaling pathways in K63-dependent cascades but not K48-dependent proteosomal degradation pathway. The rationale for the hypothesis was originated from our previous observation that proteasome inhibition paradoxically enhanced proinflammatory cytokine signaling in human astrocytes [22]. We further demonstrated that the inhibitory effect of mutant ubiquitin is mainly attributed to decreased activity of UPS-independent function of ubiquitin by computational modeling and simulation [34]. It has also been proposed that UBB^+1^ ubiquitination at K63 might exert harmful effect by enhancing NF-κB activity and subsequent NF-κB-mediated inflammatory processes [31]. Recently, it has been shown that unanchored K63 polyubiquitin chains can regulate multiple cellular signaling including direct modulation of the NF-κB pathway [33]. Our current observation did not support the proinflammatory role of UBB^+1^ in the CNS by showing anti-inflammatory action. Even though we have clearly demonstrated that UBB^+1^ can affect the cellular signaling in proteasome-independent manner, the precise molecular mechanisms underlying inhibitory action should be investigated.

Alternative action of UBB^+1^ has also been proposed for pathological degeneration of neurons in the AD brains [35]. Mutant ubiquitin causes extensive neuritic beading of mitochondria in association with neuronal degeneration probably due to inefficient axonal transport. For normal neuronal activity, mitochondria should be transported and exchanged to and from the axonal ends. Therefore, suppression of mitochondria transport can affect the neuronal activity. Recently, mitochondrial dynamics have been extensively studied for the role of differentiation and degeneration of neurons and other types of cells in the CNS [36]. Mitochondrial dynamics can be controlled by sets of proteins involving mitochondrial fission and fusion, and mitophagy [37], [38]. These proteins can also be dynamically regulated by the UPS-dependent and UPS-independent mechanisms. We are now investigating the potential role of UBB^+1^ in mitochondrial dynamics in various cell types and its functional significance.

Sustained inflammation is the hallmark of the AD brains mainly occurring in the pathologically vulnerable areas accompanied by activation of microglia and astrocytes and release of proinflammatory cytokines such as TNF-α and IL-1β. The concomitant activation of glial cells and secretion of inflammatory mediators constitute a deleterious positive feedback loop contributing to neuronal dysfunction and eventually cell death. Therefore, the inhibitory effect of UBB^+1^ on cytokine signaling might be beneficial in terms of neuroinflammation and neurodegeneration. However, the expression of UBB^+1^ can affect cellular functions via multiple mechanisms other than anti-inflammation. The overall outcome of UBB^+1^ should be reconsidered in the context of balance between tissue damage and accompanying inflammation.
